# A novel filtering T-shaped transmission line structure and its application in the design of reduced-size Butler matrix

**DOI:** 10.1038/s41598-026-44962-y

**Published:** 2026-03-26

**Authors:** Yerassyl Amangeldi, Dinesh Rano, Sultangali Arzykulov, Mohammad Hashmi

**Affiliations:** 1 Department of Electrical and Computer Engineering, School of Engineering and Digital Sciences, Nazarbayev University, Astana, Kazakhstan; 2 Department of Electrical and Electronics Engineering, Birla Institute of Technology and Science, Pilani, Pilani Campus, Vidya Vihar, Pilani, Rajasthan India

**Keywords:** Butler matrix, Phased array, Analog beamforming, Compact, Filtering, Microstrip technology, Size reduction, Engineering, Physics

## Abstract

This work presents a novel T-shaped transmission line structure designed for physical size reduction. It is analytically demonstrated that, for a specific operation frequency, the proposed structure behaves exactly like a λ/4 transmission line. Furthermore, the open-ended part of the structure can be tuned for controlled frequency rejection, thus exhibiting a built-in low-pass filtering behavior. The applicability of the network is illustrated through a practical design of the miniaturized 4 × 4 Butler Matrix (BM). As a result, the obtained BM is reduced in size by 65% compared to the classic implementation and is also capable of low-pass filtering. The theoretical and simulated return loss, insertion loss, and phase difference are in close agreement with the corresponding measured values, which proves the effectiveness of the proposed design. The designed BM has been further investigated for practical applicability as a beamforming network using patch array antennas in a simulation environment. The designed antenna array system achieves a maximum gain of 10 dBi in boresight with a possible output steering angle of ±40°.

## Introduction

There have been a number of developments in the transmission line based circuits in recent past^1–7^. In general, the use of such transmission line advancements have been utilized in the design and investigation of various Radio Frequency (RF) circuits and systems, such as antenna, MIMO systems, rectifier etc^8–16^. Among these, the Butler Matrix (BM) finds wide-ranging use in wireless power transfer, radar systems, and effective beamforming in 5G systems^11^. Several reports in the literature provide evidence of improvements in BM performance in terms of steering angle, bandwidth, and configurability in the number of ports^17–20^. However, their relatively larger physical size negatively impacts the final applications of the BM. For BM’s miniaturization, compact realization can be achieved through the division of the layout into separate vertical layers connected through vias^21–23^. However, this method is beset with lead to inferior performance, thereby limiting its effectiveness.

To reduce the physical dimensions of the BM, some authors have also proposed decreasing the number of subcomponents applied in BM realization by utilizing couplers with arbitrary power division and phase distribution^24–28^. Consequently, the obtained BM layouts do not contain crossovers or phase shifters and are made of modified couplers only. Although such solutions provide a complete analytical description and possess low complexity during fabrication, it is constrained to $4 \times 4$ BM topology. Specifically, there is no clear way of applying this approach to other BM configurations. In^21,22,27^, main subcomponents of BM, such as microstrip hybrid couplers, crossovers, and phase shifters, are replaced with patch and slotted alternatives that are implemented with a smaller physical size. As a result, miniaturized BM is obtained from their assembly. Although this approach greatly reduces the physical size, there is no clear analytical procedure for their theoretical design. In other words, this type of solution is difficult to optimize or design for different practical specifications. Similarly to the previous techniques, some authors proposed the replacement of the transmission lines applied in every subcomponent of the BM with artificial transmission lines^25,28^. This single-layer approach effectively minimizes the physical layout, but it is highly complex analytically; therefore, it is challenging to adjust for other design-specific conditions.

**Fig. 1:**
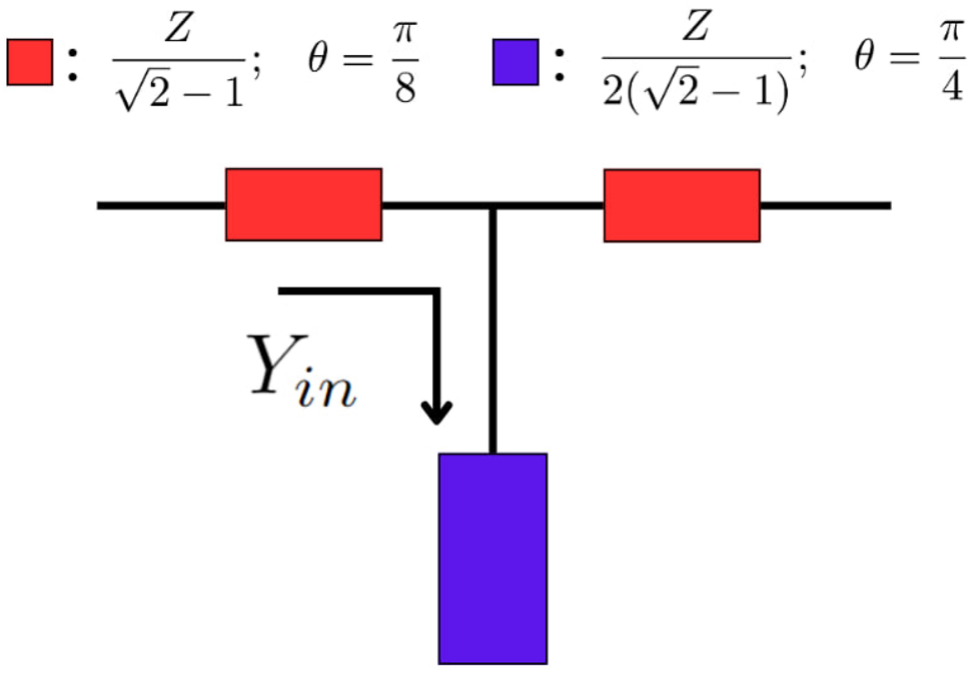
Proposed structure.

This paper, therefore, presents a novel single-layer transmission line structure, which is simple, that can be effectively applied to reduce the physical size of BM. Furthermore, the shunt-connected part can be tuned to achieve controllable low-pass filtering functionality, preserving the desired performance at the operational frequency. To support the achieved performance, a comprehensive analytical description of the structure is derived through the transmission parameters of the overall network, encompassing both normal operation and filtering. Based on these analytical derivations, the structure is utilized to implement the subcomponents of BM—hybrid couplers and crossovers. Then, they are assembled into a complete layout of 4 × 4 BM, which is prototyped and tested for expected practical operation. Furthermore, a simulation-based phased array experiment of the developed BM has been provided, which highlights the applicability of the designed BM as well as of the proposed structure for miniaturization.

The orientation of the paper is as follows: Section "Proposed design and analysis" introduces the design methodology of the BM. In addition, the filtering functionality of the proposed design, miniaturization aspects of crossover, and hybrid coupler are also presented in this section. Section "Results" presents the prototyping of the proposed design and a performance comparison of the simulation results with that of the measured one. Furthermore, in this section, a phased array is designed using the proposed BM and gain is simulated for all the output ports. Finally, sect. "Conclusion" concludes the paper.

## Proposed design and analysis

### Operation frequency performance

**Fig. 2:**
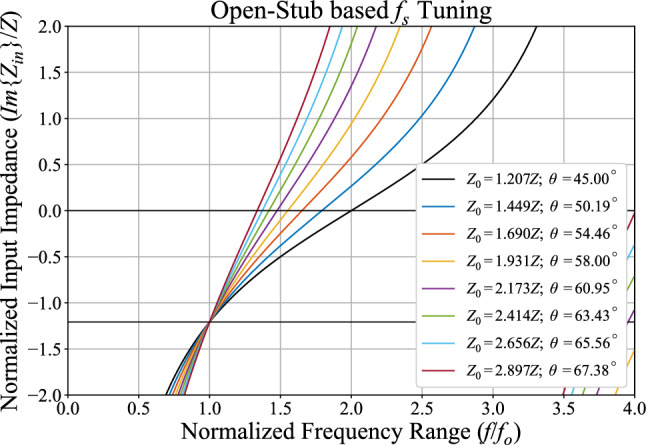
Change in $f_s$ based on the different open stubs.

Figure 1 presents the layout of the structure for an operation frequency $f_o$, where *Z* denotes the design impedance. To analyze the performance of the network, transmission parameters of the open-ended stub are identified first, as given in (1)^29^. Then, the transmission parameters of the overall T-section are obtained by consecutive matrix multiplication and simplification as given in (2). Thus, it is evident that the structure’s transmission parameters are equivalent to the respective parameters of the $\lambda$/4 transmission line with characteristic impedance equal to *Z* at the same $f_o$. Furthermore, since the electrical length of the $\lambda$/4 transmission line at the $f_o$ is equal to $\frac{\pi }{2}$, the proposed network occupies half of the horizontal space compared to it. Although the application of the network introduces the need for vertical space, this disadvantage can be negated by a clever microstrip layout (Fig. 2).

1 $$\begin{aligned} \left. \begin{bmatrix} 1 & 0 \\ Y_{in} & 1 \end{bmatrix} = \begin{bmatrix} 1 & 0 \\ j{\frac{2(\sqrt{2}-1)}{Z\cot (\theta )}} & 1 \end{bmatrix} \right| _{{\theta = \frac{\pi }{4}}} = \begin{bmatrix} 1 & 0 \\ j{\frac{2(\sqrt{2}-1)}{Z}} & 1 \end{bmatrix} \end{aligned}$$

2 $$\begin{aligned} {\left. \left( \begin{bmatrix} {\cos \theta } & j\,{\dfrac{Z}{\sqrt{2}-1}\sin \theta } \\ j\,{\dfrac{\sqrt{2}-1}{Z}\sin \theta } & {\cos \theta } \end{bmatrix} \begin{bmatrix} 1 & 0 \\ Y_{in} & 1 \end{bmatrix} \begin{bmatrix} {\cos \theta } & j\,{\dfrac{Z}{\sqrt{2}-1}\sin \theta } \\ j\,{\dfrac{\sqrt{2}-1}{Z}\sin \theta } & {\cos \theta } \end{bmatrix} \right) \right| _{\theta =\pi /8} = \begin{bmatrix} 0 & jZ \\ \dfrac{j}{Z} & 0 \end{bmatrix} } \end{aligned}$$

### Filtering functionality

It can be demonstrated using (1) that for $\theta$ equal to $\frac{\pi }{2}$ or at a frequency equal to 2$f_o$, the open-ended part of the proposed network exhibits infinite input admittance. Therefore, the input power cannot reach the output port due to the direct connection to the ground. Consequently, the network stops transmitting the signal, exhibiting low-pass filtering behavior.

Furthermore, the stopband frequency ($f_s$) of the structure can be controlled by replacing the open-ended part with an appropriate shunt component that provides the input admittance, as given in (3).

3 $$\begin{aligned} \begin{matrix} f_o: Y_{in} = j{\frac{2(\sqrt{2}-1)}{Z}} \\ \\ f_s: Y_{in} \rightarrow \infty \end{matrix} \end{aligned}$$

**Fig. 3:**
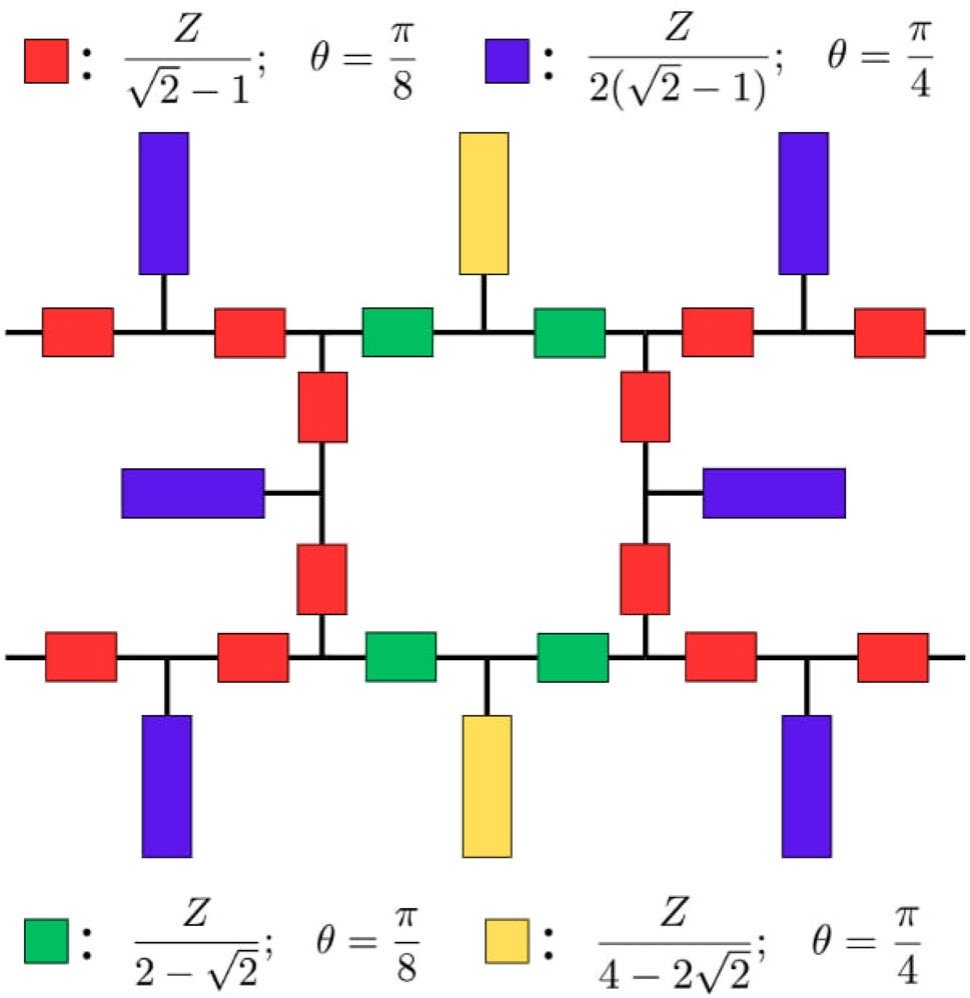
Modified filtering hybrid coupler.

This can be achieved by using another open stub, or two-section cascaded transmission lines, as in^30^. For instance, Fig. 3 demonstrates a set of open stubs that exhibit the same input impedance required for the proper operation of the structure at $f_o$, while possessing different resonance points that can be utilized for $f_s$ control. The characteristic impedance is expressed in terms of design impedance, and electrical lengths are defined at $f_o$.

Despite the overall resemblance of the proposed network to a special case of a stub-loaded resonator (SLR), it should be emphasized that the structure operates based on different principles. SLRs generally rely on the tunability of the overall even- and odd-mode admittance zeros along with operation at the resonance^31,]^^32^. In contrast, the tunability of the proposed T-shaped network is associated only with the open-ended section as described in (3), whereas the remaining part of the structure cannot be freely adjusted to preserve the $\lambda$/4 transmission line operation at $f_o$

To illustrate the capabilities of the proposed network, a $4 \times 4$ low-pass filtering Butler Matrix (BM) has been designed with a cut-off frequency of 4 GHz. To design the reduced-size filtering BM, the structure will be used to replace the $\lambda$/4 transmission lines from classical topologies of the BM’s subcomponents.

### Reduced-size filtering hybrid coupler

The classical layout of the hybrid coupler is modified through a series of replacements with the proposed network to achieve the desired reduction in size. Consequently, the theoretical layout of the new coupler is developed as in Fig. 3.

Regarding the coupler’s physical realization, the transmission lines with the least characteristic impedance, approximately equal to 0.854*Z* and with the highest characteristic impedance of around 2.414*Z*, colored in yellow and blue, respectively, are both within the realizable range for standard 50 $\Omega$ design impedance.

Since the structure is implemented to replace the input and output ports, its filtering performance will propagate to the overall device, thus creating a filtering hybrid coupler with reduced dimensions. To demonstrate the controllability of the filtering functionality, the open-ended parts of the structure at the input will be tuned according to (3) to produce complete isolation at around 4 GHz.

### Reduced-size filtering crossover

**Fig. 4:**
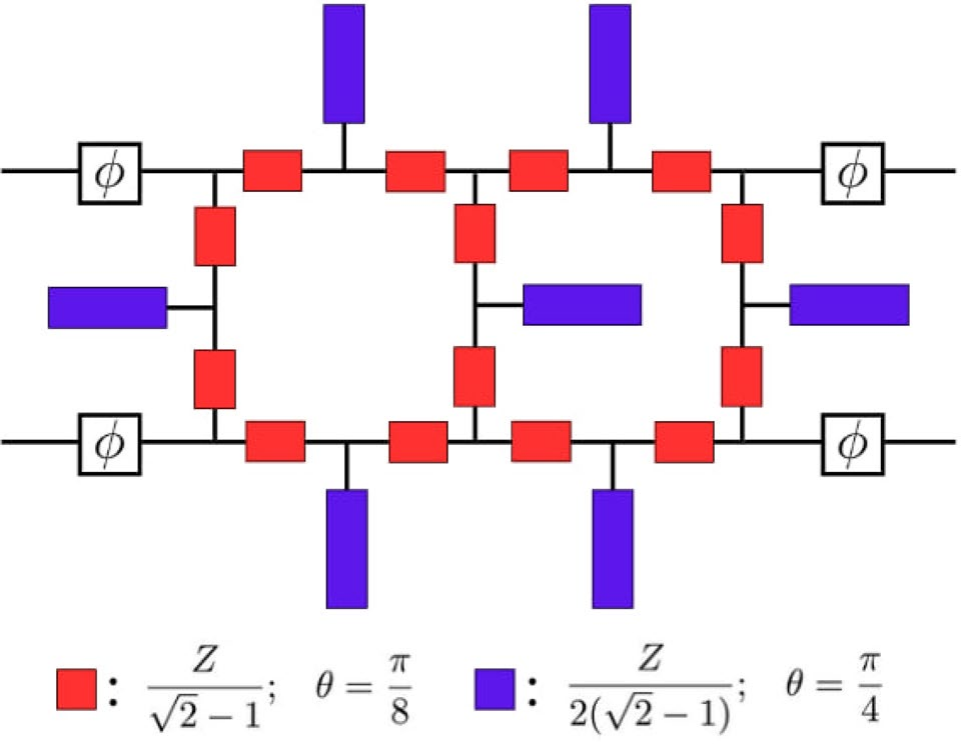
Modified filtering crossover.

Similar to the hybrid coupler, the microstrip crossover has been modified by applying the proposed network to achieve physical miniaturization, as depicted in Fig. 4. Due to the phase-shift mismatch in the classic realization, phase shifters have been introduced at the input ports to compensate for it. The maximum and minimum values of the characteristic impedance of the involved transmission lines are approximately 2.414*Z* and 1.207*Z*, respectively. Thus, the actual values fall within the range of realizable standard design impedance. Since the signal from each BM’s input port first passes through the hybrid couplers, the hybrid couplers’ filtering functionality is sufficient to implement the filtering in the final BM layout. However, the open-ended sections of the crossover reinforce the final BM’s frequency rejection.

Lastly, despite the advantages of the proposed approach for size reduction and filtering integration, it has important limitations that should be addressed. Firstly, the network can replace only $\lambda$/4 transmission lines; thus, it can be applied to systems that primarily consist of such lines to achieve greater miniaturization. Secondly, the filtering functionality obtained is controlled solely by the resonance of the structure’s open-ended part. Therefore, other important filter characteristics, such as roll-off, passband smoothness, and group delay, cannot be controlled explicitly by the proposed network. Finally, the network is capable of $\lambda$/4 transmission line’s performance at a single frequency. In other words, the expected performance of a $\lambda$/4 transmission line can be guaranteed around the operation frequency only.

## Results

**Fig. 5:**
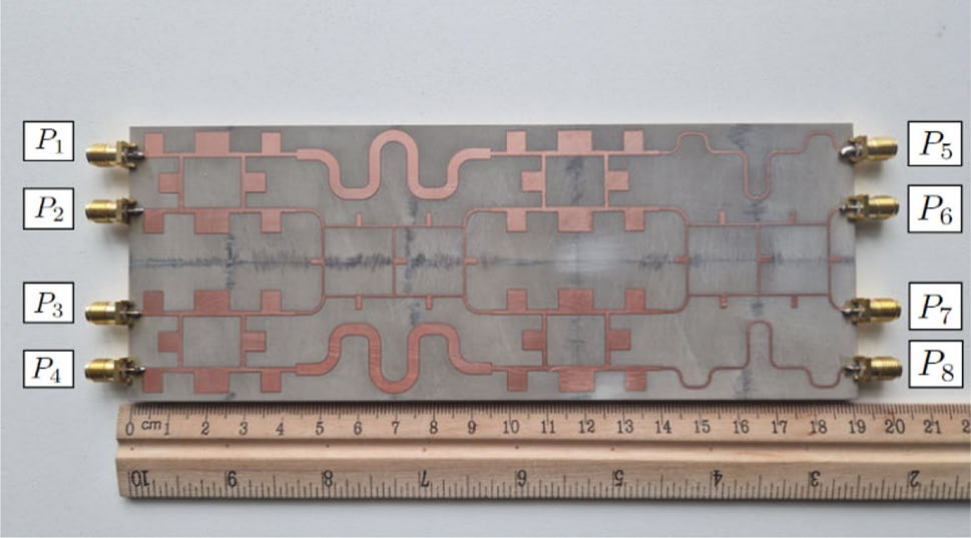
Developed BM setup.

### Developed BM setup

Based on the developed components in sect. "Proposed design and analysis" and the classic topology, a $4 \times 4$ microstrip BM layout is designed on Roger 4003C board of height 1.524 mm to operate at 2.5 GHz, as shown in Fig. 5. To achieve the required phase differences at the output ports, the necessary 45$^\circ$ phase shifters are developed and connected between hybrid couplers from different stages. Additionally, output ports 5 and 8 have been extended by 360$^\circ$ transmission lines to be placed on the same level as output ports 6 and 7. Practical measurements are limited to ports 1 and 2, considering the symmetry of the developed BM.

**Fig. 6:**
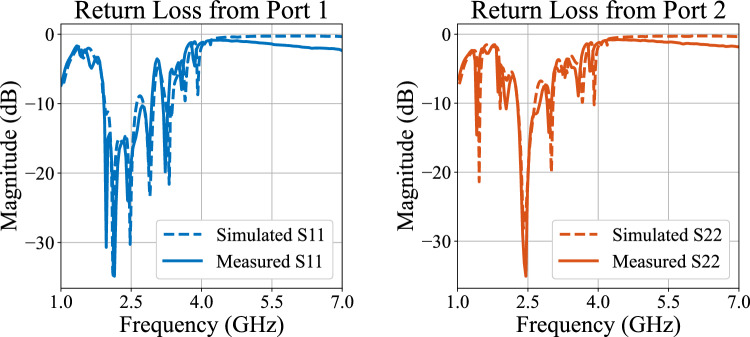
Return loss from input ports of the developed BM setup.

**Fig. 7:**
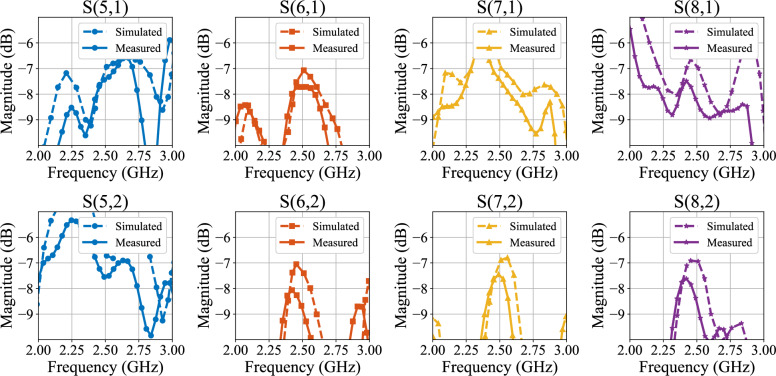
Insertion loss to output ports around $f_o$ of the developed BM setup.

The measured return loss from input ports achieves 24 dB and 34 dB at the operational frequency for port 1 and port 2, respectively, as depicted in Fig. 6. Furthermore, transmission to output ports from the first port equals − 7 dB on average with a 0.9 dB maximum imbalance between $S_{71}$ and $S_{81}$, as shown in Fig. 7. Similarly, insertion loss from port 2 is approximately − 8 dB on average with a 1 dB difference between the maximum and minimum value among output ports. Table 1 lists the phase difference between adjacent output ports, which aligns well with the ideal and the simulated values. However, a slight shift in the phase values of measured and simulated results is apparent from Table 1. The observed deviation between the simulated and measured results can be attributed to several practical factors. Specifically, the influence of SMA connectors applied during measurements has not been reflected in the simulation results due to the software’s limitations. Additionally, important substrate parameters, such as dielectric constant and microstrip insertion loss, may deviate from the manufacturer’s reported values, potentially significantly affecting the practical performance of the fabricated prototype. Despite this, the expected (ideal/simulated) and practical performance closely match, which justifies the effectiveness of the proposed design. Moreover, the return loss plots (both measured and simulated) in Fig. 6 indicate a high level of reflection of the input signal above 4 GHz, demonstrating the low-pass filtering functionality.

**Table 1:** Phase differences between output ports.

	Ideal	Simulated	Measured
$\angle S_{61} - \angle S_{51}$	$-45^\circ$	$-42.762^\circ$	$-41.231^\circ$
$\angle S_{71} - \angle S_{61}$	$-45^\circ$	$-43.287^\circ$	$-43.453^\circ$
$\angle S_{81} - \angle S_{71}$	$-45^\circ$	$-44.852^\circ$	$-45.357^\circ$
$\angle S_{62} - \angle S_{52}$	$135^\circ$	$134.751^\circ$	$133.867^\circ$
$\angle S_{72} - \angle S_{52}$	$135^\circ$	$132.254^\circ$	$131.961^\circ$
$\angle S_{82} - \angle S_{72}$	$135^\circ$	$137.955^\circ$	$138.544^\circ$

### Developed phased array

**Fig. 8:**
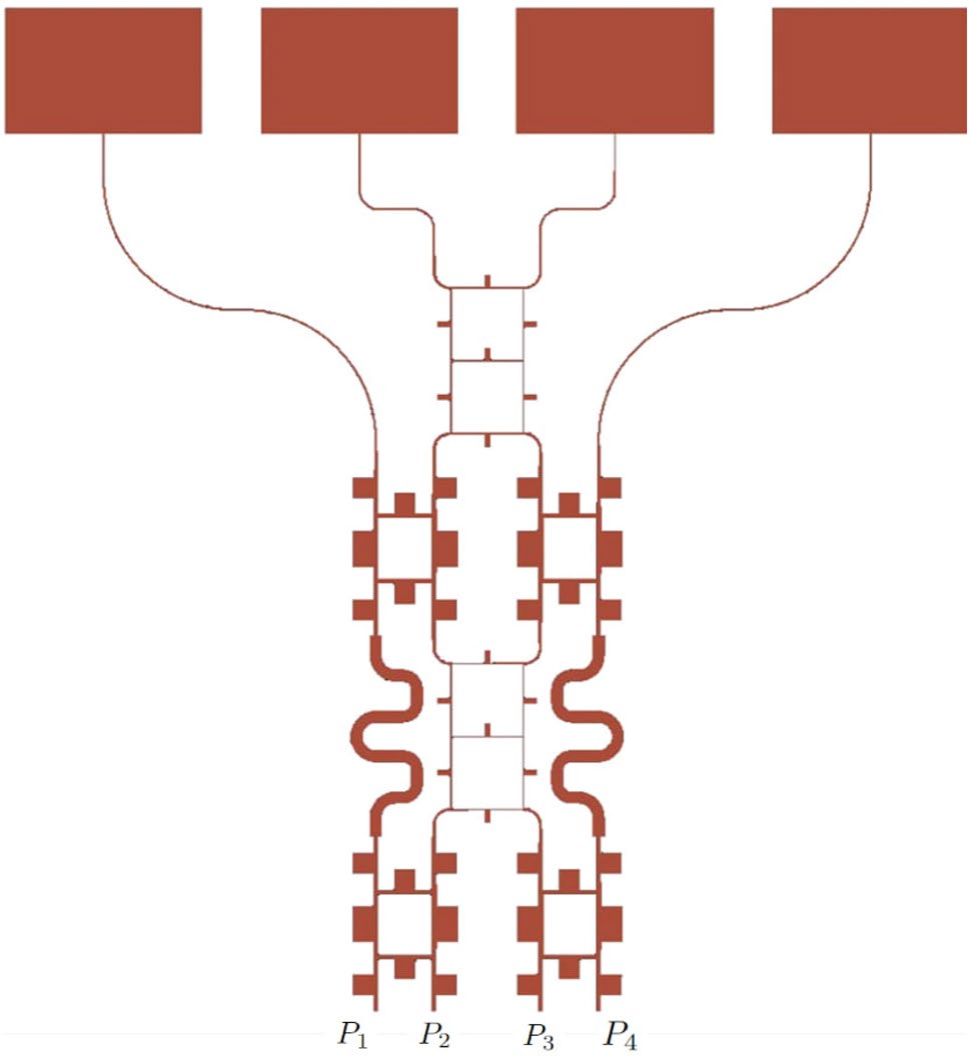
Developed BM setup with antenna array.

**Fig. 9:**
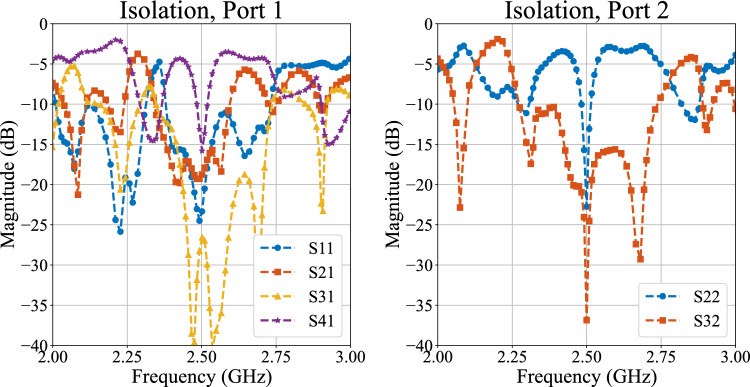
Simulated isolation between input ports.

To validate the practical applicability of the proposed reduced-size BM, a phased array has been designed using rectangular patch elements. As a result, an analog beamforming circuitry is obtained by connecting the miniaturized BM’s output ports to array elements via transmission lines with equal phase shift, as shown in Fig. 8. The simulated isolation of more than − 15 dB between the ports, 1–2 (− 20 dB), 1–3 (− 30 dB), and 1–4 (− 17 dB) of the beamforming system is apparent from the plots in Fig. 9. A slightly lower isolation between the ports is due to higher coupling between the antenna array elements arranged in E-plane geometry^33^. In addition, the input impedance of applied antennas does not exhibit exactly 50$\Omega$ input impedance at $f_o$, which is another factor of higher coupling between the elements. The bandwidth of the overall system also decreased significantly compared to BM’s separate performance due to the respective inherited property (high Q factor) of the patch antennas^33^.

**Fig. 10:**
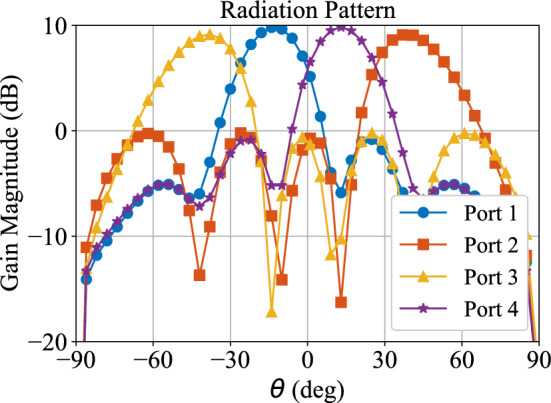
Simulated radiation pattern at $f_o$.

Figure 10 depicts the simulated far-field radiation pattern of the obtained array at $f_o$. The directions of the major lobes are complementary between Port 4 and Port 1, which is equal to ±15$^\circ$ respectively. Similarly, the largest steering angles are obtained from the excitation of Port 2 and Port 3, which are approximately ±40$^\circ$, respectively. The far-field boresight gain is approximately 10 dBi for Ports 1 and 4, while it is approximately 9.5 dBi for Ports 2 and 3. The level of the far-field sidelobes in the radiation pattern is below − 5 dBi regardless of the excited port. As a result, a developed BM exhibits an appropriate and expected performance of the classic BM’s physical realization, but with approximately one-third of its original area consumption.

Finally, Table 2 presents a comparison of the proposed BM with several state-of-the-art works in terms of size, fractional bandwidth (FBW), filtering function, complexity, insertion loss imbalance (ILI), and phase imbalance (PDI). It is apparent from Table 2 that, in terms of most of the performance parameters mentioned, the proposed design values are substantially superior to those of previous works.

**Table 2:** Comparison of developed $4 \times 4$ BM with reported designs.

Reported designs	Size ($\lambda ^2_o$)	$f_o$ (GHz)	FBW	Total layers	Filtering behavior	Fixed to number of I/O ports	Analytical complexity	ILI (dB)	PDI ($^\circ$)
^21^	3.49$\times$2.20	29	13.8%	5	No	No	Great	±1.4	N/A
^22^	0.79$\times$1.23	2.4	8%	2	Yes	Yes	Medium	±0.7	±10
^23^	3.21$\times$3.20	25.5	8%	5	No	Yes	Simple	±1.1	±4
^24^	1.08$\times$1.13	2.56	45.3%	1	No	Yes	Simple	±1.0	±7
^25^	2.30$\times$1.42	2.4	33.3%	1	No	No	Medium	±1.8	±13
**This work**	**0.97**$\times$**2.63**	2.5	19%	**1**	**Yes**	No	**Simple**	±0.5	±3

## Conclusion

To conclude, a simple filtering T-shaped structure has been developed for $4 \times 4$ BM miniaturization purposes. A theoretical explanation of the structure’s working principles is provided analytically. Moreover, the network incorporates filtering functionality via the shunt-connected part’s resonance, which can be adjusted to specific frequencies. The essential aspect of the proposed design is its simplicity in analytical and practical implementation compared to other works. Its operation can be explained in terms of transmission parameters, and it does not require additional layers for size reduction. Moreover, it is versatile in application because it does not depend on the specific BM layout and can be applied to other microwave systems that contain $\lambda$/4 transmission lines. Finally, the technique can be easily applied in conjunction with other reported methods to yield greater size reduction, thereby improving the final cost of beamforming realization in the context of 5G.

## Data Availability

The datasets generated and/or analysed during the current study are not publicly available due to confidentiality of the work, but are available from the corresponding author on reasonable request.
